# *In Vivo* Islet Protection by a Nuclear Import Inhibitor in a Mouse Model of Type 1 Diabetes

**DOI:** 10.1371/journal.pone.0013235

**Published:** 2010-10-06

**Authors:** Daniel J. Moore, Jozef Zienkiewicz, Peggy L. Kendall, Danya Liu, Xueyan Liu, Ruth Ann Veach, Robert D. Collins, Jacek Hawiger

**Affiliations:** 1 Department of Microbiology and Immunology, Vanderbilt University Medical Center, Nashville, Tennessee, United States of America; 2 Department of Pediatrics, Ian Burr Division of Endocrinology and Diabetes, Vanderbilt University Medical Center, Nashville, Tennessee, United States of America; 3 Department of Medicine, Vanderbilt University Medical Center, Nashville, Tennessee, United States of America; 4 Department of Pathology, Vanderbilt University Medical Center, Nashville, Tennessee, United States of America; 5 Department of Surgery and Emory Transplant Center, Emory University, Atlanta, Georgia, United States of America; University of Bremen, Germany

## Abstract

**Background:**

Insulin-dependent Type 1 diabetes (T1D) is a devastating autoimmune disease that destroys beta cells within the pancreatic islets and afflicts over 10 million people worldwide. These patients face life-long risks for blindness, cardiovascular and renal diseases, and complications of insulin treatment. New therapies that protect islets from autoimmune destruction and allow continuing insulin production are needed. Increasing evidence regarding the pathomechanism of T1D indicates that islets are destroyed by the relentless attack by autoreactive immune cells evolving from an aberrant action of the innate, in addition to adaptive, immune system that produces islet-toxic cytokines, chemokines, and other effectors of islet inflammation. We tested the hypothesis that targeting nuclear import of stress-responsive transcription factors evoked by agonist-stimulated innate and adaptive immunity receptors would protect islets from autoimmune destruction.

**Principal Findings:**

Here we show that a first-in-class inhibitor of nuclear import, cSN50 peptide, affords *in vivo* islet protection following a 2-day course of intense treatment in NOD mice, which resulted in a diabetes-free state for one year without apparent toxicity. This nuclear import inhibitor precipitously reduces the accumulation of islet-destructive autoreactive lymphocytes while enhancing activation-induced cell death of T and B lymphocytes derived from autoimmune diabetes-prone, non-obese diabetic (NOD) mice that develop T1D. Moreover, in this widely used model of human T1D we noted attenuation of pro-inflammatory cytokine and chemokine production in immune cells.

**Conclusions:**

These results indicate that a novel form of immunotherapy that targets nuclear import can arrest inflammation-driven destruction of insulin-producing beta cells at the site of autoimmune attack within pancreatic islets during the progression of T1D.

## Introduction

Type 1 diabetes (T1D) results from the progressive destruction of insulin-producing beta cells in pancreatic islets caused by pro-inflammatory and pro-apoptotic effectors of innate and adaptive immunity. Extraordinary advances with insulin monotherapy and understanding of the critical role of the adaptive immune system in the T1D pathomechanism have not translated to diabetes reversal. Patients remain at risk for the serious complications inherent to the autoimmune and metabolic derangements in T1D. Given the side effects of insulin therapy and current immunosuppressive regimens, the search for new therapeutic approaches continues [1]. The requisite roles of islet-specific autoreactive T and B cells have been well established and have been the primary target of current clinical investigations [2], [3], [4], [5]. Building on the role of adaptive immunity, both T cell-directed immunotherapy with anti-CD3 and the B cell-directed action of rituximab (anti-CD20) have shown similar efficacy in delaying the progression of new-onset diabetes [2], [6]. Unfortunately, while clinical benefit to patients in these trials has been recorded[7], insulin-secreting capacity continues to decline in treated individuals and these regimens have not restored stable tolerance to islet tissue, perhaps because they do not completely target the islet-destructive autoimmune inflammatory process.

A growing body of evidence now suggests that the innate immune system, which is antigen non-specific and tightly coupled to acute and chronic inflammation, plays an equally important role in diabetes progression in genetically-predisposed individuals and may also be amenable to therapeutic intervention [8], [9], [10], [11], [12]. Clinically, the presence of chronic inflammation is suggested by the persistent elevation in C-reactive protein and also in the propensity of individuals with T1D to develop accelerated atherosclerosis, which is now viewed as an inflammatory disorder, irrespective of glycemic control [13], [14]. Mechanistically, the key role of innate immunity has been further supported by the recent report that genetic ablation of the key adaptor of innate immunity, MyD88, affords protection from T1D in specific pathogen-free NOD mice [15]. Indeed, this study documented that MyD88 signaling is critical for development of T1D; thus, MyD88-dependent activation of innate immune cells by non-tolerogenic constituents of the intestinal microbiome may be an initiating event in the development of insulitis, the inflammatory hallmark of T1D [16], [17].

With respect to Type 1 diabetes progression, pro-inflammatory signaling initiated through stimulation of the principal receptors of innate immunity—Toll-like receptors (TLRs)—is one mechanism that activates antigen-presenting cells (APCs). In turn, these effectors of innate immunity render effector T cells resistant to regulatory T cell (Treg)-mediated suppression [18], [19]. As a consequence, loss of peripheral tolerance ensues. This loss is consistent with reports that naïve T cells in NOD mice are resistant to Treg action [20], [21]. Given their escape from both peripheral and central selection processes, autoreactive T and B cells go on to produce critical pro-inflammatory cytokines TNF-α, IL-1β, and IFN-γ that can lead directly to beta cell programmed cell death (apoptosis) [22].

Production of these islet-toxic cytokines depends on tightly-regulated intracellular signal transduction by stress-responsive transcription factors (SRTFs), such as NF-κB, AP-1, NF-AT, STAT-1, and others. NF-κB is the paradigmatic SRTF and is well-known for its role in diabetes pathogenesis with crucial roles played at the levels of both lymphocytes and beta cells [23], [24], [25]. However, other SRTFs, including NF-AT, AP-1, and STAT-1, have also been implicated [26], [27], [28] by activating numerous target genes that encode mediators of inflammation and apoptosis, which underlie destruction of islets and other target tissues. These positive effectors of pro-inflammatory immune signaling to the nucleus participate in an auto-stimulatory loop, which amplifies the inflammatory process initiated by microbial and autoimmune triggers. Thus, uncontrolled nuclear translocation of SRTFs represents an additional feature of the dysregulated immunity of the murine model of Type 1 diabetes that may disrupt peripheral tolerance [29], [30], [31], [32], [33].

To carry out these potential diabetogenic functions, activated SRTFs are ferried to the nucleus of cells responding to innate and adaptive immune stimulation. This culminating step in TLR-evoked innate immunity and TCR-evoked adaptive immunity is mediated by nuclear import adaptors known as importins/karyopherins [34]. Thus, targeting these cytoplasmic adaptors with an inhibitor of nuclear import offers a new level of control toward dysregulated innate and adaptive immune signaling in T1D.

In this report, we present evidence for a new mode of T1D control that allows simultaneous inhibition of TLR-evoked innate immunity and TCR-initiated adaptive immunity. Since both immune responses depend on intracellular signal transduction by stress responsive transcription factors, we targeted the nuclear import mechanism with a first-in-class, cell-penetrating nuclear import inhibitor. We found that short-term intracellular delivery of this inhibitor afforded long-term protection of the islets from inflammation-driven apoptosis. This long-lasting (one year) islet-protecting effect, which arrests diabetes progression without the need for insulin therapy, appears to involve the precipitous reduction of autoreactive lymphocytes through enhancement of activation-induced cell death of T and B lymphocytes. Moreover, this salutary effect of short-term nuclear import targeting is associated with reprogramming of the pro-inflammatory and anti-inflammatory cytokine profile of immune cells isolated from NOD mice.

## Results

### Primary lymphocytes and macrophages from NOD mice are susceptible to attenuation of T cell receptor (TCR)- and Toll-like receptor (TLR)-evoked pro-inflammatory signaling by a nuclear import inhibitor

**Figure 1:**
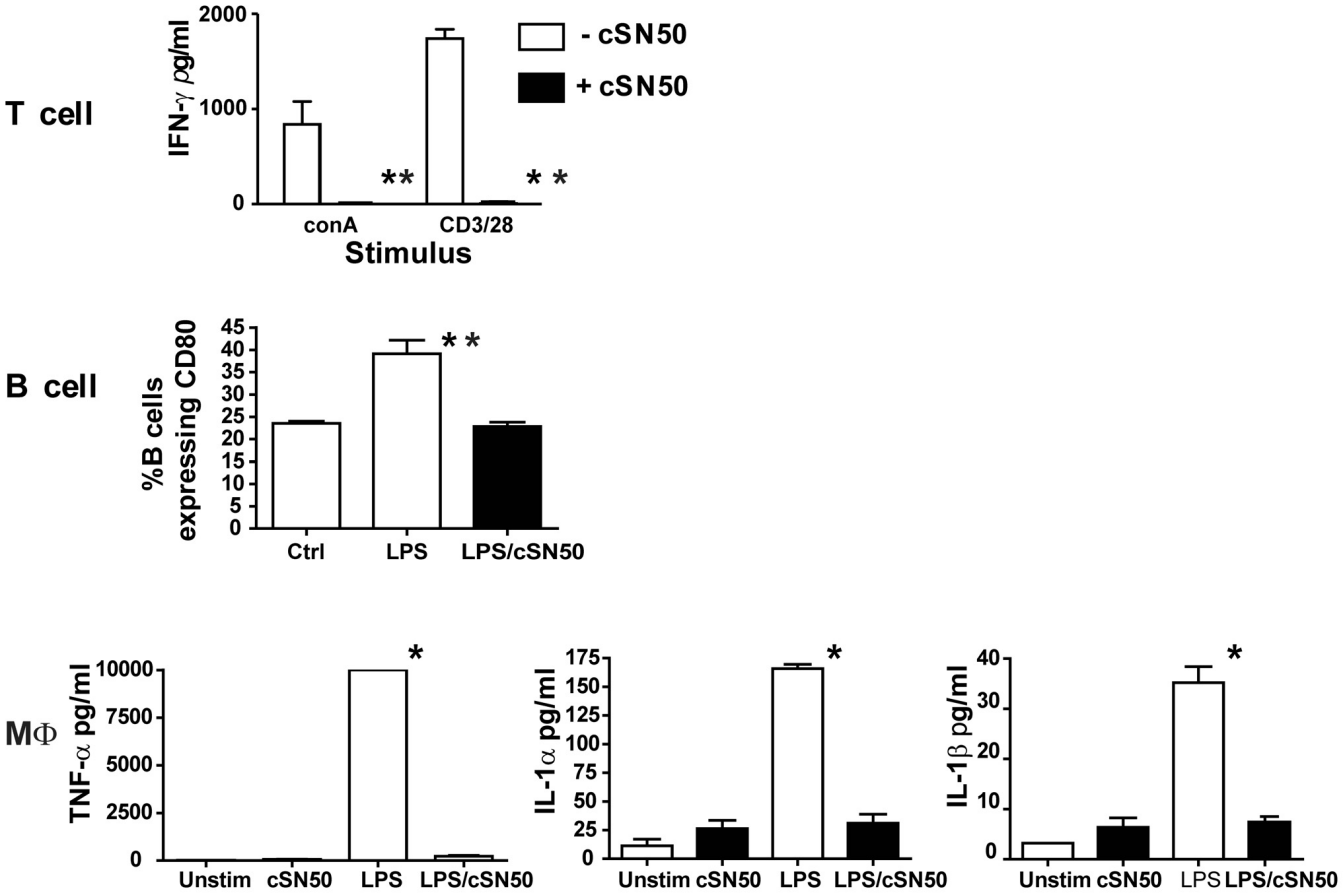
Nuclear import inhibitor suppresses both T Cell Receptor- and Toll-Like Receptor-evoked signaling. Splenocytes from 10 week old NOD females were isolated and stimulated with anti-CD3/CD28 (2 µg/ml), concanavalin (1 µg/ml) (conA), or LPS (5 µg/ml) in the presence or absence of nuclear import inhibitor (cSN50 peptide at 30 µM). Supernatants were harvested at 72 h and analyzed for presence of (**A**) IFNγ as a measure of T cell activation using cytokine bead array (**p<0.01 vs cSN50, t-test) and (**B**), Up-regulation of CD80 (B7.1) as a measure of B cell responsiveness was assessed by flow cytometry (**p<0.01, Student's t-test) on cells co-expressing CD19 as a B cell marker. (**C**) Pro-inflammatory cytokine production in bone marrow derived-macrophages cultured in L-conditioned media. Differentiated cells were stimulated with LPS (10 ng/ml) in the presence or absence of cSN50 (30 µM). cSN50 inhibited production of TNF-α, IL-1α, and IL-1β (*p<0.05, Student's t-test). Data is representative of three or more experiments.

NOD mice, a widely used model of human T1D, demonstrate genetic predisposition toward autoimmune diabetes as evidenced by numerous immunologic abnormalities of innate and adaptive immunity as compared to normal strains of mice [12], [15], [30], [31], [35]. We had previously demonstrated that nuclear import inhibitor, cSN50 peptide, attenuates TCR- and TLR-evoked cytokine/chemokine production in murine models of acute inflammation and apoptosis using normal C57BL/6 and BALB/c strains [36], [37], [38], [39]. Therefore, we first examined whether agonist-stimulated T and B lymphocytes and macrophages derived from autoimmune diabetes-prone NOD mice are suppressed by targeting nuclear import with cell-penetrating cSN50 peptide (**Fig. 1**). Accordingly, we stimulated isolated NOD splenocytes with the T cell agonists anti-CD3/CD28 or concanavalin A (Con A) in the presence or absence of the nuclear import inhibitory peptide cSN50 and measured the production of the pro-apoptotic cytokine IFN-γ. cSN50 suppressed the robust production of this islet-toxic cytokine. Furthermore, we assessed the effect of cSN50 on B cells and bone marrow-derived macrophages (BMDM) prepared from NOD mice and stimulated with the pro-inflammatory agonist LPS that is recognized by Toll-like receptor (TLR) 4 on macrophages and B cells. As NOD-derived B lymphocytes are hyper-responsive to LPS in terms of CD80 (B7.1) expression [35], we demonstrated that this response is attenuated by cSN50. Moreover, expression of islet-toxic cytokines TNF-α, IL-1α, and IL-1β in BMDM obtained from NOD mice was suppressed. Thus, a nuclear import inhibitor attenuates production of islet-toxic, pro-apoptotic mediators evoked by TCR and TLR agonists in primary immune cells derived from NOD mice.

### Intracellular delivery of a nuclear import inhibitor to the pancreas reduces islet inflammation (insulitis)

**Figure 2:**
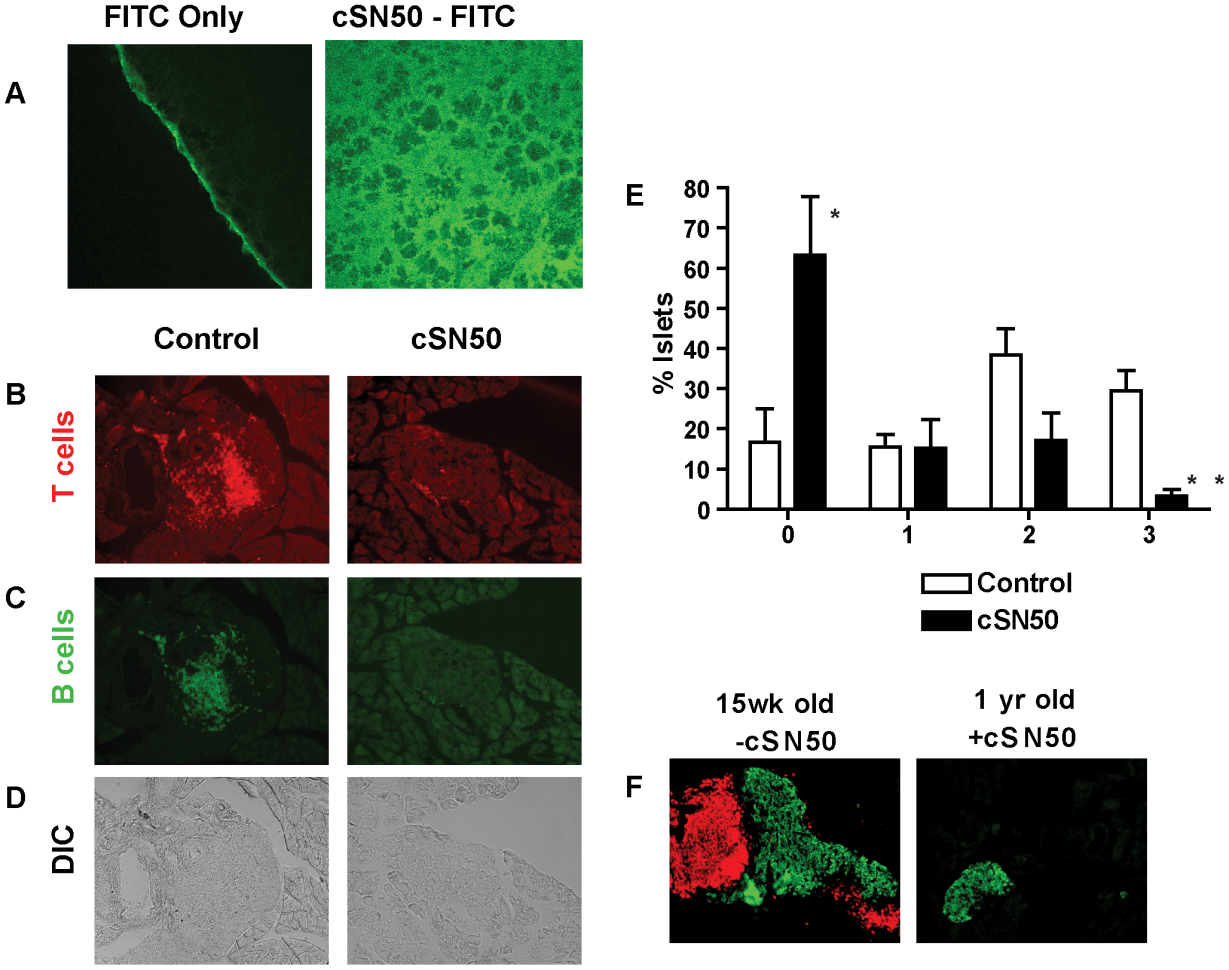
*In vivo* intracellular delivery of a nuclear import inhibitor to the pancreas attenuates insulitis. (**A**) cSN50 was conjugated to FITC per the manufacturer's instruction. Animals received one i.p. injection of FITC-cSN50 or an amount of FITC of equivalent relative fluorescence. Animals were euthanized after 2 h and 10 µm frozen sections were cut and assessed by confocal microscopy. The cSN50-FITC peptide is distributed throughout the pancreas. In contrast, FITC alone did not penetrate the organ and only the autofluorescent tissue border is seen. Pancreas is shown at 40x magnification. (**B–D**) cSN50 therapy attenuates ongoing insulitis. 10 week-old NOD females received one injection of Cy (0.2 mg/g). 45 h after this injection, treatment was begun with cSN50 (35 µg/g every two hours) and continued for 24 h at which time the seven animals in each group (treatment and control) were euthanized and pancreata obtained. Sections were stained for (**B**) CD3-PE (red) and (**C**) B220 (green). The differential interference contrast (DIC or Nomarski) image is shown in panel (**D**). Of 7 animals assessed in each group, four NOD mice receiving cSN50 were completely insulitis free as compared to persistent insulitis in all control group animals (p<0.05, chi-square). (**E**) All NOD mice receiving histologic evaluation in cSN50-treated and control groups (n = 7 for each) were assigned insulitis scores based on the severity of inflammation (0 = no invading cells, 1 = peri-insulits, 2 = invasive insulitis, 3 = minimal residual islet tissue). Comparison of control to cSN50- treated animals shows significantly more unaffected islets in treated mice (*p<0.05) and significantly fewer severely affected islets (**p<0.001). (**F**) Three animals were assessed at 1 year follow-up for insulin production and insulitis. Sections were stained with a combination of anti-insulin FITC (green) and a combination of anti-CD3 and anti-B220PE (red). In long-term survivors (right), insulin staining is detected but insulitis was not observed. As a staining control, islets from 15 week old control NODs show significant insulitis.

The highly effective suppression of pro-inflammatory and pro-apoptotic cytokines in *ex vivo* analysis of primary, NOD-derived immune cells following treatment with cSN50 peptide encouraged us to embark on *in vivo* study of Type 1 diabetes in NOD mice. We first determined whether cSN50 would be delivered to the pancreas, the primary site of autoimmune attack against beta cells in pancreatic islets. We have demonstrated previously that this cell-penetrating nuclear import inhibitory peptide is delivered to blood leukocytes/lymphocytes, spleen, liver, and lung to suppress acute liver and lung inflammation [36], [38], [40]. Using confocal microscopy, we assessed the pancreatic delivery of FITC-labeled cSN50 peptide following a single intraperitoneal (i.p.) injection. Rapid and uniform intracellular delivery of FITC-cSN50 was apparent throughout the pancreas within 2 h after injection (**Fig. 2A**).

Given this finding, we investigated the effect of cSN50 delivery on the ongoing insulitis. We selected the well-characterized accelerated model of autoimmune diabetes in the NOD mouse following a single bolus of cyclophosphamide (Cy), which synchronizes progression of T1D. In this widely used animal model of human Type 1 diabetes, two to four days following Cy injection a peak pro-inflammatory cytokine response is reported, which is followed by development of overt autoimmune diabetes in 2–4 weeks [41], [42], [43], [44]. Therefore, we challenged 10-week old female NOD mice with Cy in two groups of seven. We initiated treatment with cSN50 or control (either a non-cell penetrating peptide denoted cN50 or saline) 45 h later. To assure a steady level of nuclear import inhibitor in blood and pancreas (**see** **Fig. 2A**), we adopted a high intensity treatment protocol by administering 35 µg/g of cSN50 i.p. every two hours for the next 24 h; mice receiving control peptide received a molar equivalent. We analyzed pancreatic sections by immunohistochemistry (**Fig. 2B–D**). Strikingly, 57% of treated mice following one-day therapy with cSN50 were free of insulitis while significant insulitis remained in all age-matched controls receiving Cy alone (p<0.05 vs treated). Our control finding compares well with other reports of insulitis at this age which find nearly 100% of NOD mice to demonstrate insulitis affecting a significant number of pancreatic islets [45]. Islets from both groups of animals were assigned insulitis scores based on the severity of inflammation (**Fig. 2E**). Comparison of control to cSN50- treated animals shows significantly more unaffected islets in cSN50-treated mice (p<0.05) and significantly fewer severely affected islets (p<0.001). Mean insulitis score was also determined and showed a value of 0.49 in the cSN50-treated group and 1.81 in the control group (p = 0.001, Mann-Whitney U test). One year after the short-course treatment, three treated mice were also analyzed histologically. All three of these animals continued to show no signs of insulitis (**Fig. 2F**). Thus, a short-term intracellular delivery of cSN50 to the pancreas is followed by a rapid reversal of ongoing insulitis that is otherwise exacerbated in untreated NOD mice when T1D is synchronized with Cy.

### Islet-reactive T cells are reduced and activation-induced cell death (AICD) is enhanced by intracellular delivery of a nuclear import inhibitor

**Figure 3:**
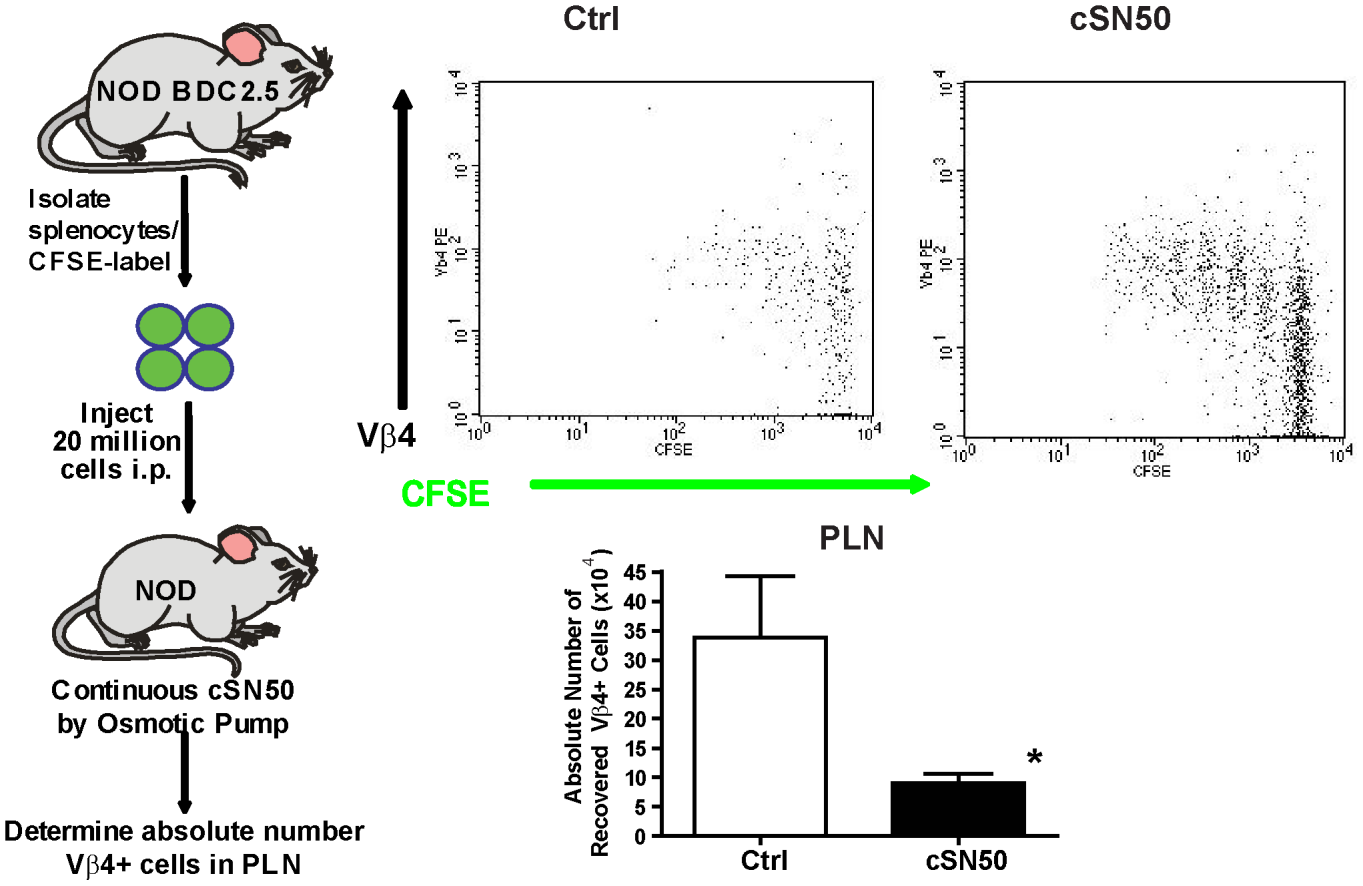
Autoreactive T cells disappear following *in vivo* treatment with a nuclear import inhibitor. Twenty million CFSE- labeled BDC2.5 splenocytes were transferred to pre-diabetic NOD recipients who received cSN50 peptide or saline by osmotic pump. On day 4 post-transfer, pancreatic lymph node cells were harvested and the proliferation profile of CD4+Thy1.1- cells and absolute number of CFSE+ Vβ4+ cells within the CD4 compartment was determined. Treated mice showed significant reduction in the absolute number of recovered Vβ4+ cells (*p<0.05, student's t-test). Figures are representative of three separate experiments.

**Figure 4:**
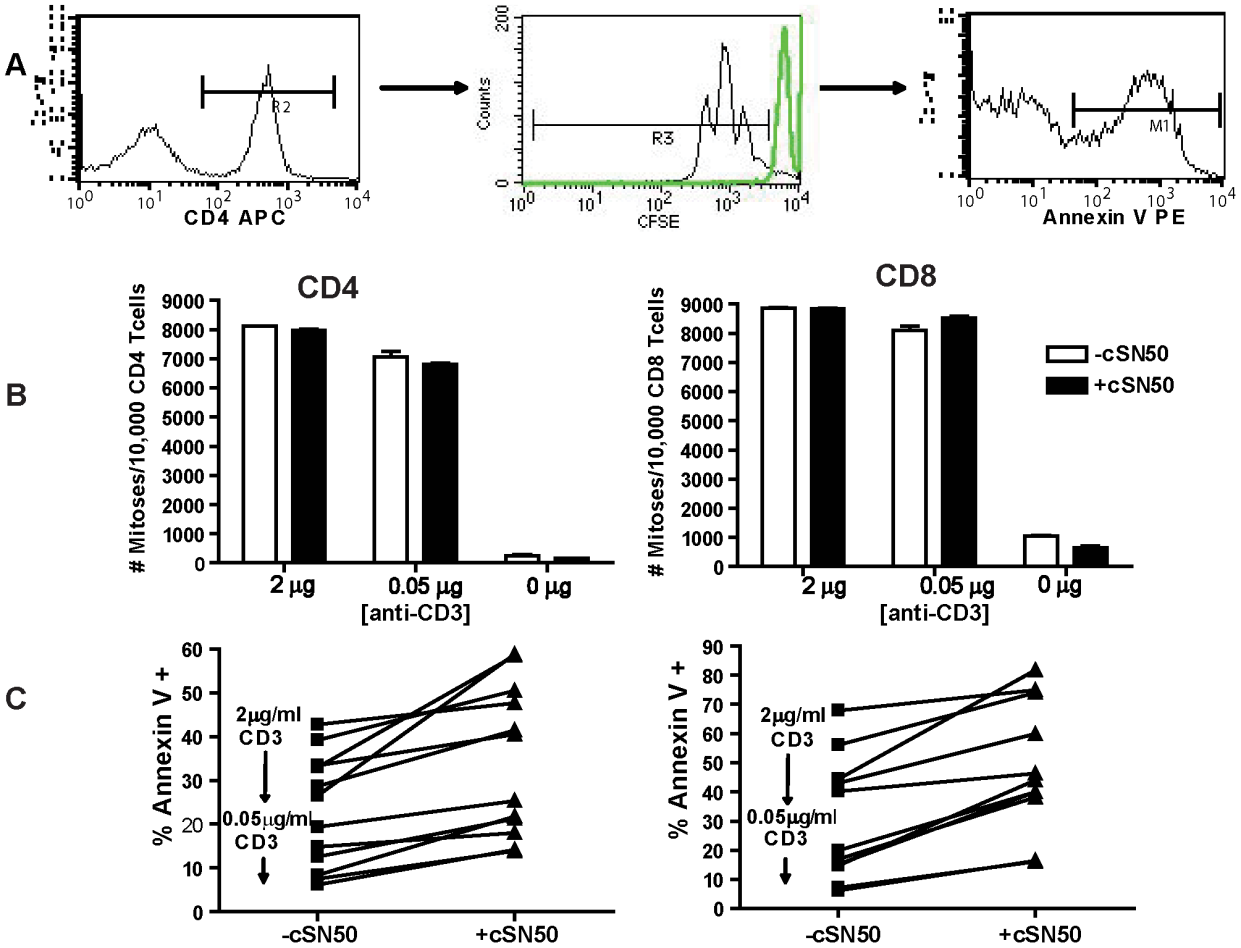
Activation-induced cell death (AICD) is enhanced following *ex vivo* intracellular delivery of a nuclear import inhibitor to splenocytes. Splenocytes from 10 week- old NOD females were labeled with CFSE and stimulated with anti-CD3 (0.05 µg/ml or 2 µg/ml) and anti-CD28 (1 µg/ml) in the presence or absence of cSN50 (30 µM). After 65 h of culture, samples were harvested and labeled with annexin V-PE, anti-CD8 PerCP, and anti-CD4 APC. For analysis of AICD, activation was defined as the achievement of at least one round of division as determined by CFSE-dilution; a representative gating strategy is shown in (**A**); the undivided peak is highlighted in green and derived from unstimulated cells in the same experiment. (**B**) The CFSE division history, represented graphically as the number of mitoses per 10,000 CD4 or CD8 T cells, demonstrated no change in proliferation in CD4 or CD8 T cells in the presence of cSN50 (p = NS). (**C**) Further analysis revealed increased annexin-V staining on activated cells, indicative of cellular apoptosis, for cSN50-treated CD4 and CD8 T cells (p<0.005, paired student's t-test). Data is representative of at least 4 separate experiments.

**Figure 5:**
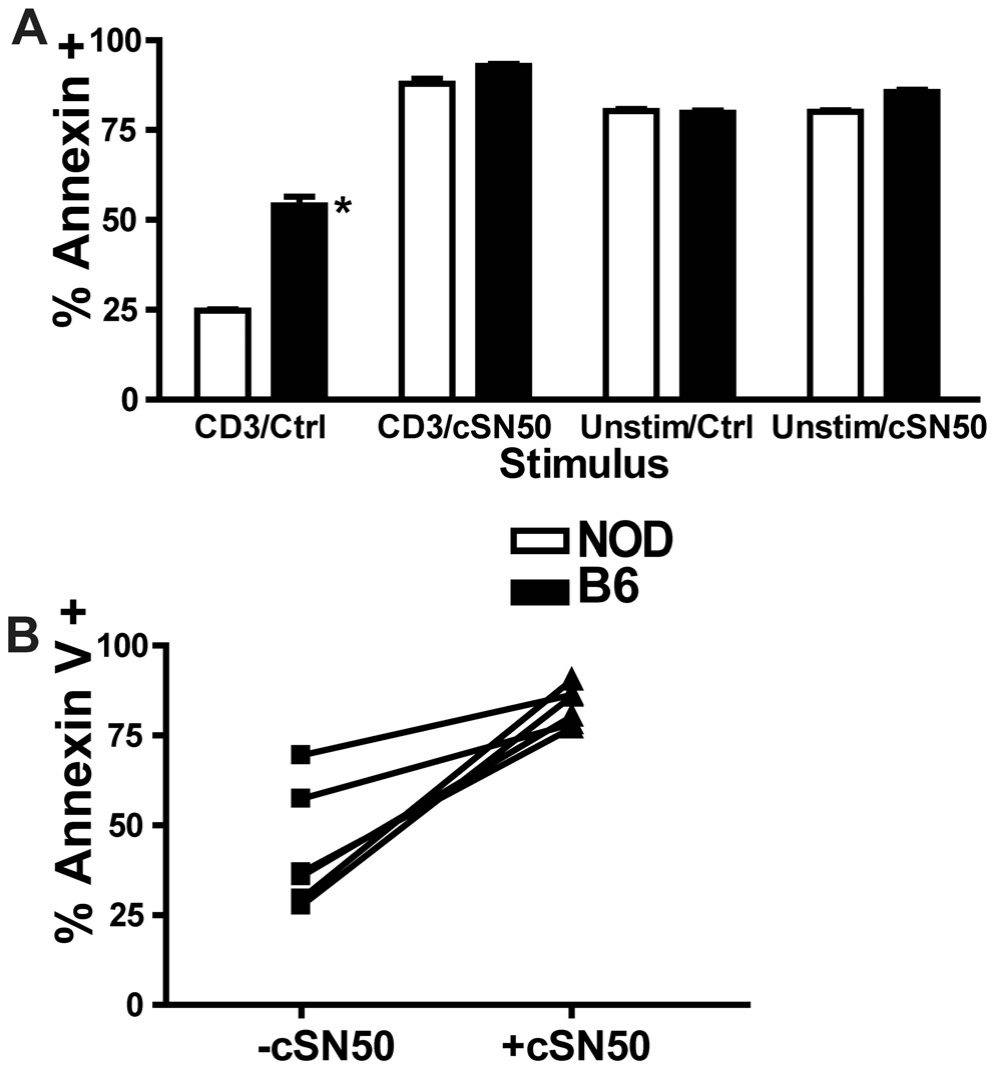
cSN50 restores Activation-Induced Cell Death (apoptosis) in NOD CD4 T cells and enhances B cell apoptosis in response to LPS. Splenocytes from 10-week-old NOD or C57BL/6 female mice were isolated and stimulated with anti-CD3/28 (2 µg/ml) or 5 µg/ml LPS in the presence or absence of cSN50 (30 µM). After 70 h culture, cells were harvested and apoptosis was detected by annexin V PE staining with lymphocyte co-labeling with either anti-CD4 APC (**A**) or anti-CD19 APC (**B**). (**A**) NOD splenocytes when stimulated with anti-CD3/28 showed reduced apoptosis as compared to C57BL/6 splenocytes as previously published (*p<0.01, t-test). Addition of cSN50 normalized apoptosis to C57BL/6 levels (p = NS). Unstimulated lymphocytes showed high levels of apoptosis, as expected for unactivated lymphocytes in culture; this process was not enhanced by cSN50. (**B**) Exposure to cSN50 significantly enhanced sensitivity to cell death in LPS-stimulated B lymphocytes (p<0.005, paired Student's t-test).

Ongoing insulitis, as a hallmark of T1D in NOD mice, is driven in part by the persistence of activated, autoreactive T and B lymphocytes. They are resistant to activation induced cell death (AICD), a critical mechanism for the loss of peripheral T cell tolerance [30], [31], [32], [33]. Having observed elimination of T- and B-lymphocytes in the islets of cSN50-treated NOD mice (**Fig. 2B and 2C**), we hypothesized that intracellularly delivered cSN50 may enhance lymphocyte sensitivity to AICD, thereby reversing the known resistance of NOD lymphocytes to this process [30], [32], [33]. Although we had not observed changes in total lymphocyte numbers above the transient cell loss following Cy exposure (data not shown), we considered that autoreactive cells may have been depleted following intense short-term treatment with the nuclear import inhibitor. Therefore, we utilized adoptive transfer of islet-reactive BDC2.5 lymphocytes to test their persistence and expansion in pancreatic lymph nodes during treatment with the nuclear import inhibitor. Donor cells were labeled with CFSE and transferred to recipients that received cSN50 or saline continuously via osmotic pump for 4 days (**Fig. 3**). Analysis of the pancreatic lymph node demonstrated a striking reduction in transferred islet-reactive cells in the cSN50- treated mice, as demonstrated by a significant decrease in the absolute number of CFSE-labeled, Vβ4+ cells recovered at the end of treatment. Islet-reactive BDC2.5 lymphocytes were precipitously reduced following cSN50) peptide treatment either by an effect on cell proliferation, lymphocyte survival, or lymphocyte entry to the node. We directly assessed the effect of the nuclear import inhibitor on cell survival and proliferation by analyzing the sensitivity of NOD lymphocytes to AICD in an *ex vivo* assay. Splenocytes were harvested from 10 week old NOD females and stimulated with a defined concentration of anti-CD3/CD28 for 65 h in the presence or absence of cSN50 before detection of apoptosis with annexin-V staining (**Fig. 4A**). Importantly, we focused on the detection of apoptosis in NOD lymphocytes with definitive evidence of activation as determined by the achievement of at least one cell division. Use of CFSE to calculate the number of mitotic events following anti-CD3/CD28 stimulation revealed that cSN50 had no effect on CD4 or CD8 T cell proliferation (**Fig. 4B**). In contrast, intracellular delivery of cSN50 increased the sensitivity to AICD in CD3/CD28-stimulated CD4+ and CD8+ T cells (**Fig. 4C**). As anticipated, CD4 T cells from NOD mice were generally more resistant to AICD but following cSN50 peptide delivery, they displayed a significant increase in sensitivity to AICD; in fact, the percentage of CD4 T cells undergoing AICD reached values comparable to T cells obtained from the non-diabetes prone C57BL/6 strain (**Fig. 5A**). In parallel to the significant change in sensitivity to AICD noted in NOD mice-derived T cells, B lymphocytes also had an increase in sensitivity to apoptosis induced by their agonist in the presence of cSN50 peptide. When B cells were analyzed with similar methods following stimulation with the TLR4 agonist, LPS, they displayed enhanced propensity for cell death (**Fig. 5B**). Thus, the nuclear import inhibitor not only reduced the number of islet-reactive T cells but restored sensitivity to AICD in T and B lymphocytes isolated from NOD mice.

### Immunomodulatory Cytokines IL-5 and IL-10 are enhanced in animals treated with a nuclear import inhibitor

**Figure 6:**
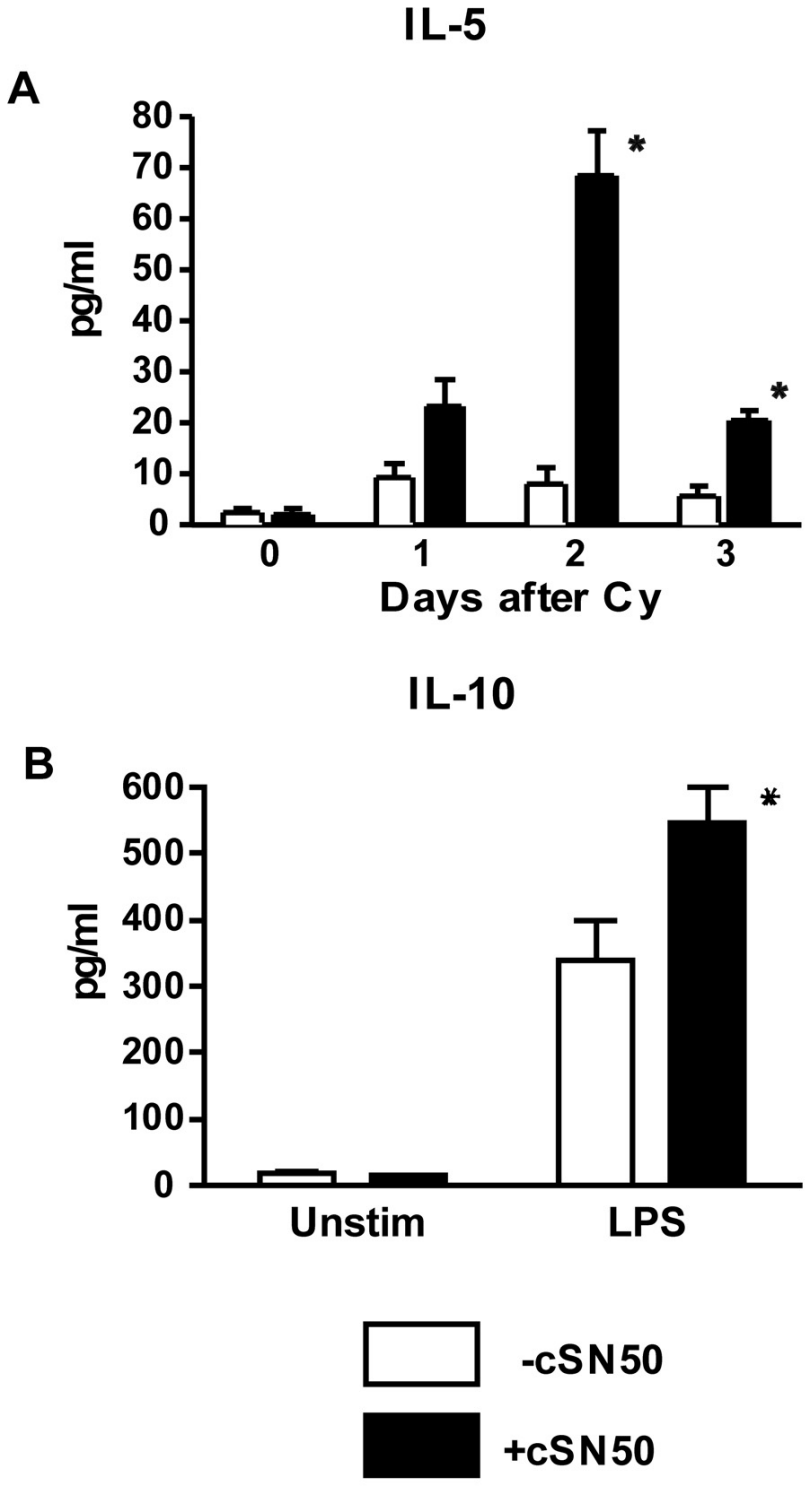
Expression of immunomodulatory cytokines IL-5 and IL-10 is modified during treatment with a nuclear import inhibitor. (**A**) Blood samples were obtained daily by saphenous vein bleeding beginning on the day of Cy challenge. Cytokine levels in plasma were determined by cytokine bead array and comparison to a standard curve. cSN50-treated mice demonstrate increased IL-5 (p<0.05 ANOVA; subsequent analysis of individual days by Student's t-test shows *p<0.05 for days 2 and 3) during therapy. (**B**) Splenocytes from 10 week-old NOD females that had received cyclophosphamide were harvested after 24 hr of treatment with cSN50 or control non-cell-penetrating peptide (cN50) and restimulated with LPS to assess their cytokine profile. Supernatants were harvested at 72 h and analyzed by cytokine bead array for presence of IL-10. Splenocytes from cSN50 treated animals produced increased levels IL-10 (*p<0.05). Data are from three separate experiments.

Although cSN50 treatment eliminated the majority of islet-reactive lymphocytes and facilitated reduction of insulitis, these islet-reactive and invading immune cells did not completely vanish following intense 1- or 4- day delivery of cSN50 peptide via ip injection or osmotic pump, respectively. Therefore, we considered whether there had been augmentation in regulatory cells or whether the remaining lymphocytes demonstrate alteration in their cytokine expression profile. Analysis of the absolute number of regulatory T cells was determined by intracellular Foxp3 staining and showed no difference between treated and control mice at either the end of treatment (48 h) or two weeks after treatment concluded. With respect to cytokine expression, the systemic effect of cSN50 on cytokines/chemokine production in blood of NOD mice was monitored daily in plasma samples in treated and control mice beginning on the day of Cy administration until 12 days following peptide treatment (day 14 post- Cy bolus). During this period of administration, we detected increased circulating levels of IL-5 on day 2 after Cy administration (Fig 6a). No differences were detected in plasma levels of TNF-α, IFN-γ, IL-10, IL-12, IL-2, or IL-4, Eotaxin, GM-CSF, IL-1α, IL-1β, M-CSF, IL-3, IL-7, IL-9, IL-12 (p40), IL-12 (p70), IL-13, IL-15, IL-17, IP-10, MIP-2, KC, LIF, LIX, MIP-1α, MIP-1β, MIG, RANTES, or VEGF (data not shown) and most of these markers of inflammation were barely detectable in plasma or were below the limit of detection. However, we still considered that the capacity for production of anti-inflammatory cytokines may have been modified although increased systemic production of anti-inflammatory cytokines, such as IL-10, was not detected. Therefore, we obtained splenocytes from NOD females that had received 24-h peptide therapy beginning two days after Cy acceleration to examine the effect of cSN50 peptide on *ex vivo* cytokine production by immune cells. Restimulated splenocytes from treated animals showed increased production of the anti-inflammatory cytokine IL-10 suggesting that cSN50 peptide was not simply a global suppressant of pro-inflammatory cytokine/chemokine production in NOD mice but rather a modulator of lymphocyte function in lymphoid organs (**Figure 6b**). Together with results depicted in Figure 1, these cumulative data indicate the ability of a nuclear import inhibitor to exert a bimodal effect on primary NOD immune cells by suppressing the expression of pro-inflammatory mediators, including islet-toxic cytokines like IFN-γ and TNF-α while preserving or enhancing anti-inflammatory cytokines such as IL-10.

### Short-term treatment with nuclear import inhibitor prevents accelerated T1D in NOD mice during a one-year observation

**Figure 7:**
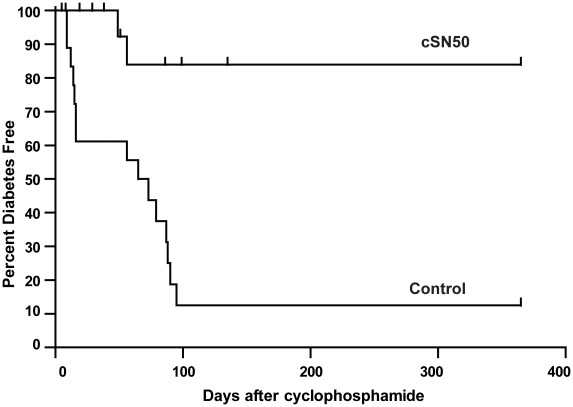
Short-term intracellular delivery of a nuclear import inhibitor *in vivo* protects NOD mice from autoimmune diabetes for over one year. Ten week old female NOD mice received one dose of Cy (0.2 mg/g) to synchronize autoimmune diabetes progression. After 45 h, intracellular peptide delivery was initiated with cSN50 (35 µg/g) or with control and continued every 2 h for 48 h. Blood glucose was assessed twice weekly. cSN50-treated mice (n = 20) were significantly protected from diabetes progression as compared to saline-treated control (n = 10, p<0.0001) or the non-cell-penetrating peptide cN50-treated control (n = 10, p = 0.006). A comparison of cSN50- treated vs. the combined control groups as illustrated in the figure also demonstrated significant protection (p = 0.0002, log-rank test).

**Figure 8:**
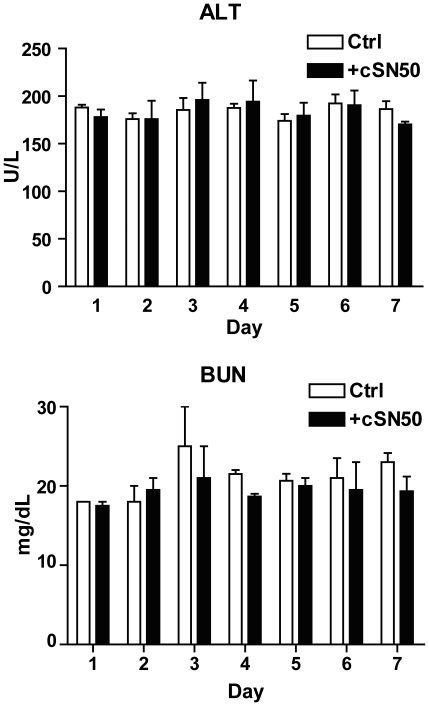
Short-term treatment of NOD mice with a nuclear import inhibitor shows no effect on measured clinical biomarkers of liver and kidney toxicity. Ten week old female NOD mice received one dose of Cy (0.2 mg/g); after 45 h, intracellular peptide delivery was initiated with cSN50 (35 µg/g) or with control cN50 and continued every 2 h for 48 h. Serum was obtained daily from the saphenous vein and measurements of ALT and BUN were performed in 7 mice in each group. No significant differences were determined. Measurements for alkaline phosphatase, creatinine, creatine kinase, and total bilirubin were below the limit of detection in both groups.

Having established a striking effect of *in vivo* delivery of cSN50 peptide on reversal of insulitis through reduction of islet-reactive T and B cells, we embarked on a pre-clinical study of cSN50 peptide in terms of its effect on T1D progression. As above, we synchronized T1D development in 10-week old female NOD mice by a single bolus of Cy. After 45 h, we used a high intensity treatment protocol by administering 35 µg/g of cSN50 i.p. every two hours for the next 48 h to assure a steady level of nuclear import inhibitor in blood and pancreas; control mice received saline or control peptide at molar equivalent. As documented in **Fig. 7**, the majority of NOD mice (90%) were rendered diabetes-free following this short-term treatment of only 2 days with cSN50. In contrast, 90% of control mice developed diabetes by 100 days after Cy bolus. The progression to diabetes in control group proceeded in two phases. On days 10–30, a first wave of diabetes developed and cSN50 afforded 100% protection against this process. In the second wave, corresponding closely to the normal progression of spontaneous diabetes between days 60–100 post-Cy (ages 18–23 weeks), protection was incomplete but still substantial even though more than two months had elapsed since the last dose of nuclear import inhibitor. Strikingly, the cSN50 peptide-treated mice remained euglycemic, which obviated the need for insulin replacement therapy. Importantly, they did not display overt signs of toxicity during 365 days of observation. The lack of toxicity of intensive treatment in this model was supported by normal weight gain, no signs of acquired infection, and normal clinical chemistries (ALT and BUN levels were not significantly different from control, **Fig. 8**). Moreover, we did not detect anti-cSN50 peptide antibody induction in an ELISA assay where the limit of detection based on a titration of cSN50-reactive IgG antibody was 10 ng/mL (O.D. values for serum were not significantly different from secondary antibody alone).

Thus, short-term targeting of the nuclear import of stress-responsive transcription factors with cSN50 peptide suppressed accelerated autoimmune diabetes progression and rendered thriving NOD mice free of diabetes progression for at least one year.

## Discussion

The ultimately fatal outcome of autoimmune diabetes in the widely used and clinically relevant murine NOD model of human T1D depends on progressive and relentless destruction of insulin-producing beta cells in pancreatic islets and is inevitable unless insulin-replacement therapy is instituted. We investigated the possibility of protecting islets from autoimmune attack by intracellular delivery of a nuclear import inhibitory peptide (cSN50). Our results indicate that cSN50 effectively protected islets from immune destruction. This protection is vested in significant reduction of islet-reactive T cells, restoration of the sensitivity of T and B cells to AICD, suppression of islet-toxic pro-inflammatory cytokine production in primary T and B cells and macrophages isolated from NOD mice, and preservation of a key anti-inflammatory cytokine, IL-10. Thus, a nuclear import inhibitor extinguished autoimmune inflammation-driven islet loss and prevented further progression of diabetes thereby obviating the need for insulin replacement therapy.

Remarkably, none of the cSN50 peptide-treated animals developed hyperglycemia during the first phase of diabetes onset occurring between days 10–30 after receiving a bolus of cyclophosphamide (Cy), which synchronized the autoimmune diabetes process in NOD mice. In addition to this early protective effect, cSN50 treatment also afforded significant long-term islet protection. While another half of Cy-synchronized control animals progressed to diabetes between days 50 and 100, only two of twenty cSN50- treated animals developed diabetes, a finding suggesting that cSN50 treatment resulted in long-term islet protection in NOD mice that are genetically-prone to T1D. This favorable outcome is supported by our demonstration of *in vivo* elimination of islet-infiltrating and islet-reactive lymphocytes (Fig. 3), most likely through enhanced AICD, which was demonstrated *ex vivo* in cSN50 peptide-treated T and B cells (Fig. 4). Our data indicate that at higher levels of stimulation (e.g., higher concentrations of the mitogenic stimulus, anti-CD3), the sensitivity to AICD is further increased by the nuclear import inhibitory peptide. Thus, chronic activation of islet-infiltrating T and B cells in autoimmune diabetes may favor the AICD-enhancing effect of the nuclear import inhibitor.

This hitherto unreported action of cSN50 in a relevant preclinical T1D model adds autoimmune inflammation to the list of conditions in which a nuclear import inhibitor has displayed therapeutic utility. We have previously demonstrated the efficacy of nuclear import inhibitor delivery and its anti-inflammatory and cytoprotective action in acute inflammation models, including lethal challenge with superantigen, staphylococcal enterotoxin B (SEB), and lipopolysaccharide (LPS), which trigger acute inflammatory lung and liver injury [40], [46]. Moreover, we have documented inhibition of nuclear entry of stress-responsive transcription factors, NF-κB, NF-AT, AP-1, and STAT-1 in human T lymphocytes [37]. Of relevance to autoimmune diabetes is the documented role of these transactivators in lymphocyte activation and beta cell apoptosis [22], [24], [47], [48], [49]. Moreover, nuclear import of Nrf2, a critical responder to oxidant stress, is also attenuated by SN50 peptide [50].

An important aspect of our nuclear import inhibitor is its ability to reach the pancreas (Fig. 2A) and cells comprising pancreatic lymph nodes, as well as other lymphoid and non-lymphoid organs. We have previously elucidated the mechanism of intracellular delivery of this peptide and documented an endocytosis-independent process of crossing the plasma membrane mediated by the membrane-translocating motif (MTM), which is based on the signal sequence hydrophobic region (SSHR) derived from Kaposi FGF [51]. The amphipatic helix-based structure of SSHR facilitates its insertion directly into plasma membrane and the tilted transmembrane orientation permits the translocation of the nuclear import inhibitor through the phospholipid bilayer of the plasma membrane directly to the interior of the cell without perturbing membrane integrity [52]. This mechanism explains the efficient delivery of SSHR-guided cargo across the plasma membrane of multiple cell types involved in autoimmune inflammation.

Overall, our study presents a new avenue for altering the course of diabetes progression as there has been limited success in obviating the need for parenteral insulin-replacement therapy of T1D to date. Recent advances in immunosuppressive/anti-inflammatory therapy using monoclonal antibodies that target T cells, B cells and cytokine receptors have produced encouraging results. These efforts have focused on targeting extracellular receptors on T and B cells without discerning islet-destructive autoreactive T and B cells from primary innate immunity cells [2], [6]. The latter encompass monocytes, macrophages, and dendritic cells that contribute not only to islet inflammation and apoptosis but also to the loss of peripheral tolerance to beta cells antigens. Consistent with their role in autoimmune inflammation, they are being also controlled by nuclear import inhibition. Hence, a broad repertoire of SRTFs-regulated genes that encode mediators of islet inflammation and beta cells apoptosis is attenuated. Contributing to the short-circuiting of this pro-inflammatory signaling cascade, nuclear import inhibition reversed resistance of autoreactive T cells to Activation-Induced Cell Death (AICD). Indeed, as islet-reactive lymphocytes are likely to be maximally stimulated during disease progression, cSN50 enhanced their deletion as compared to those lymphocytes without islet-reactive specificities (cf. Figs 2,3,5). Thus, cSN50 treatment seems to restore peripheral T and B cell tolerance, which critically depends on the appropriate regulation of lymphocyte AICD [53].

In addition to enhancing autoreactive lymphocyte elimination, the nuclear import inhibitor may also modulate the cytokine milieu established by immune cells in their target organs. We found that cSN50 inhibits pro-inflammatory cytokine expression in *ex vivo* analyzed NOD splenocytes while preserving and even modestly enhancing the anti-inflammatory cytokine IL-10. We have previously observed a similar enhancement of IL-10 in other models of acute inflammation that were ameliorated by nuclear import targeting. Moreover, we have also found an increase in IL-5 in the plasma of treated mice during the first day of cSN50 therapy. We did not find increased levels of IL-4 or IL-13 and thus it is unclear whether this increased IL-5 is indicative of a shift towards a Th2 phenotype. While Th2 shifts have occasionally been associated with diabetes protection, it is not clear that this shift is a part of the true protective mechanism in many cases [54]. Significantly, the pattern of increased IL-10 and IL-5 was also seen in human subjects in the original trial of anti-CD3 [2]. Cumulatively, these findings suggest that IL-10 and IL-5 may play an important role in modulating the course of Type 1 diabetes in tolerized individuals.

Our results with a nuclear import inhibitor suggest that reduction or complete elimination of islet-destructive autoreactive T cells may be useful in treating T1D and potentially other autoimmune diseases. Under conditions of metabolic, inflammatory, and oxidant stress, beta cell mass may be further controlled by important interactions between bone marrow-derived cells and islet beta cells [55]. Transcriptional modulation of these interactions *via* targeting of the nuclear import machinery represents an important new opportunity for therapeutic development. We are aware that the ability of cSN50 to block nuclear transport of several stress-responsive transcription factors that utilize importin/karyopherin alpha 1 for nuclear trafficking [37], [50] raises concern regarding potential side effects of this novel approach. However, intracellular delivery of the nuclear import inhibitor is rapid and restricted to the relatively short half life of the injected cargo [37]. Nonetheless, whether this therapy would be tolerated during longer-term application, if necessary, to prevent or reverse spontaneous disease remains an important consideration. It is thus reassuring that despite high intensity dosing of cSN50 in our current protocol, we did not observe short-term or long-term adverse effects. In other disease models, we have continued cSN50 peptide delivery for up to 8 weeks without evidence of toxicity including normal animal health and survival, normal weight gain, normal liver enzymes, and absence of apparent infectious illness.

In summary, this report provides the first, to our knowledge, evidence to document that short-term intensive targeting of the nuclear import shuttle for stress-responsive transcription factors can protect islets from relentless autoimmune attack and induce long-term remission of T1D in NOD mice with established insulitis. The evidence presented here that chronic inflammatory destruction of beta cells could be reversed without overt signs of toxicity should engender further preclinical studies of this novel form of intracellular immunotherapy in T1D.

## Materials and Methods

### Mice and Diabetes Monitoring

NOD/LtJ, NOD-BDC2.5 and C57BL/6 mice were purchased from the Jackson Laboratories (Bar Harbor, ME) at 6–8 weeks of age. All mice were housed and maintained according to the guidelines for use and care of laboratory animals as set forth by Vanderbilt University and regulated via the Vanderbilt IACUC. All NOD mice were monitored twice weekly for the development of diabetes by blood glucose measurement with FreeStyle FastTake test strips (Abbott Laboratories, Abbott Park, IL). Two consecutive glucose measurements >220 mg/dl constituted a diagnosis of diabetes. Although we did not maintain our own colony of NOD mice, a colony kept by a collaborating laboratory (PLK) in the same animal suite has a spontaneous diabetes incidence of 80–90% in females by 30 weeks of age [56] indicating an animal environment that is conducive for diabetes development.

### Isolation of lymphoid cells and preparation of bone marrow derived macrophages

Splenocytes and lymph node cells were prepared by dispersion of the organ and passage through a 70-µm cell strainer followed by red cell lysis and resuspension in the media of choice for the given experiment. For preparation of primary macrophages, bone marrow from pre-diabetic, 8–12 week old female NOD mice was prepared by flushing mouse femurs and tibias with ice-cold DMEM supplemented with L-glutamine. Bone marrow cells were pooled, passed through a 25 5/8-gauge needle, and filtered through a 70-µm cell strainer. Pooled cells (1×10^6^ cells/ml) were suspended in DMEM supplemented with 10% FBS, 10 mM HEPES, penicillin (100 U/ml), streptomycin (100 µg/ml), and 20% L929 conditioned medium followed by plating on 150-mm bacterial Petri dishes. Cells were incubated at 37°C in 5% CO2 in humid air. Every 3 days, non-adherent cells were removed, cells were washed, and culture medium was replaced. Cells were used in experiments after 10 days of culture for up to 2 weeks after maturation. When analyzed by flow cytometry, 95% of the adherent cells were MAC3+, CD3–, and B220–. The viability of BMDMs was >80% before use in all experiments.

### *Ex vivo* stimulation of NOD lymphocytes

Cells were plated in 24-well plates at a density of 1×10^6^ total cells/ml in DMEM containing 10% HI-FCS, penicillin (100 U/ml), streptomycin (100 µg/ml), 2-mercaptoethanol (55 µM), and varying amounts of the identified stimulus including anti-CD3 (0–2 µg/ml) with 1 µg/ml anti-CD28, LPS (5 µg/ml), or concanavalin A (1 µg/ml) (conA). All cells were incubated for 65–70 h at 37°C in 5% CO2.

### Synthesis, purification, and labeling of a cell-penetrating peptide inhibitor of nuclear import (cSN50) and its non-cell-penetrating control (cN50)

Cell-penetrating peptide (cSN50, MW = 3149), and non-cell-penetrating peptide (cN50, MW = 1651), were synthesized, purified, filter-sterilized, and analyzed as described elsewhere [37], [38]. To monitor the intracellular delivery of peptides to pancreas, cSN50 peptide was coupled with fluorescein isothiocyanate (FITC, Pierce) according to the manufacturer's protocol, as previously described [40].

### *In Vivo* Intracellular Peptide Delivery to the Pancreas

For in vivo detection of fluorescein-labeled peptides in the pancreas, NOD mice were sacrificed at 2 h after intraperitoneal (IP) injection of 0.7 mg of FITC-labeled cSN50 peptide/mouse. The pancreata were washed with saline and prepared for cryosections (10 µm thickness). Control solution of FITC alone with equivalent fluorescence units was injected separately to track distribution to the pancreas.

### Synchronization of T1D Progression with Cyclophosphamide

At 10–11 weeks of age, female NOD mice received an intraperitoneal bolus injection of 200 mg/kg cyclophosphamide (Sigma). Cyclophosphamide was prepared by reconstitution of powder in sterile saline for injection.

### Treatment with Nuclear Import Inhibitor and Control

45 h after diabetes synchronization with cyclophosphamide, treatment with cSN50 or control was initiated. Mice receiving cSN50 were given 35 µg/g of cSN50 i.p. every two hours for the next 48 h. Control mice received either saline or non-cell penetrating cN50 peptide at molar equivalent (20 µg/g). All injections were delivered in a volume of 100 µl of sterile saline

### Immunohistochemistry

Freshly harvested pancreata were fixed in 4% paraformaldehyde-0.1 M PBS (12.07 g of Na2HPO4 (dibasic), 2.04 g of KH2PO4 (monobasic), 8.0 g of NaCl, 2.0 g of KCl; pH 7.5, same-day preparation) for 1.5 h at 4°C under mild agitation, followed by four washings in 0.1 M PBS over a period of 2 h at 4°C under mild agitation. Tissue was equilibrated in 30% sucrose in 1x PBS (Invitrogen) overnight at 4°C until tissue settled to the bottom of the tube. Pancreata were then frozen in OCT (Sakura Finetek), and cut into 8 µm sections using a cryostat microtome (Leica). Sections were rehydrated with PBS for 2 min before blocking for 30 min at room temperature in blocking buffer (5% normal goat serum and 1% BSA in 1x PBS), then stained with anti-B220-FITC and anti-CD3 PE for 1 h at room temperature, washed with PBS, and mounted in fluorescent mounting medium (Dako). Slides were examined by conventional fluorescence microscopy using an Olympus BX60 epifluorescence microscope. Images were captured using a charge-coupled device camera and MagnaFire software (Optronics) and optimized for signal-to-noise using Adobe Photoshop software (Adobe Systems). A pancreas was deemed insulitis-free if at least 40 islets were identified and no islet had evidence of T or B-cell staining.

### Flow Cytometry

One million splenocytes were suspended in PBS containing 0.1% azide and 3% FCS and surface stained with the following mAbs: RM4-5 (anti-CD4), 1D3 (anti-CD19), RA3-6B2 (anti-B220), 53-6.7 (anti-CD8), or 16-10A1 (anti-CD80), each conjugated to FITC, PE, PE-Cy7, Cychrome or APC as appropriate. Annexin V staining was performed in the appropriate binding buffer as supplied. All reagents were obtained from BD Pharmingen (San Jose, CA). All samples were analyzed on FACSCalibur flow cytometer (Becton Dickinson, Mountain View, CA) using CellQuest software.

### CFSE Labeling

Spleens were harvested, and cells were labeled and prepared as previously described [30]. Mitotic events were determined as previously reported [30] based on the two-fold decrease in fluorescence intensity with each division of labeled cells.

### *In vivo* tracking of islet-reactive CD4 T cell elimination during nuclear import inhibitor treatment

Splenocytes were isolated from NOD BDC2.5 TCR transgenic mice and labeled with CFSE. A total of 20×10^6^ of these CFSE-labeled cells was injected intraperitoneally into NOD-Thy1.1 mice. For BDC2.5 T cell transfers, optimal activation occurs at about 90 h [57], and so recipient cells were harvested at this time point from pancreatic lymph nodes. Single cell suspensions were prepared and stained with anti-Thy-1.1 PE-Cy7 (OX-7), anti-TCR Vβ4-PE (the BDC2.5 transgene utilizes Vβ4 TCR) and anti-CD4 APC (RM4-5) to allow for the identification of the transferred CD4 T cells using flow cytometry. To allow continuous and facile peptide delivery throughout the 4-day incubation period, mice were implanted with osmotic pumps the day prior to cell transfer. Implantation was achieved following induction of anesthesia with ketamine and xylazine per standard protocol and the pump was placed into a subscapular location. The wound was closed with a single staple. These 4-day pumps deliver 100 ul of fluid at 1 ul/hr and generally begin infusion about 12 hrs after implantation (Alzet, Cupertino, CA).

### Cytokine detection by cytokine bead array

Serum or plasma samples were obtained daily by saphenous vein bleeding. Other samples for cytokine determination were obtained from supernatants of stimulated lymphocyte cultures. Cytokine concentration was determined by cytokine bead array and comparison to a standard curve (BD Biosciences, San Jose, CA) or with the MILLIPLEX mouse cytokine-chemokine kit (Millipore, St. Charles, MO) according to the manufacturer's specifications.

### Clinical Chemistry

ALT and BUN levels were determined on a VITROS 250 Chemistry analyzer using multi-layered vitros slides and colorimetric or enzyme rate tests as appropriate.

### Statistical analysis

Statistical comparison between groups was performed by log-rank analysis, Student's t-test or ANOVA as appropriate. A p-value less than 0.05 was considered significant.

## References

[1] Huang X, Moore DJ, Ketchum RJ, Nunemaker CS, Kovatchev B (2008). Resolving the conundrum of islet transplantation by linking metabolic dysregulation, inflammation, and immune regulation.. Endocr Rev.

[2] Herold KC, Hagopian W, Auger JA, Poumian-Ruiz E, Taylor L (2002). Anti-CD3 monoclonal antibody in new-onset type 1 diabetes mellitus.. N Engl J Med.

[3] Hu CY, Rodriguez-Pinto D, Du W, Ahuja A, Henegariu O (2007). Treatment with CD20-specific antibody prevents and reverses autoimmune diabetes in mice.. J Clin Invest.

[4] Xiu Y, Wong CP, Bouaziz JD, Hamaguchi Y, Wang Y (2008). B Lymphocyte Depletion by CD20 Monoclonal Antibody Prevents Diabetes in Nonobese Diabetic Mice despite Isotype-Specific Differences in Fc{gamma}R Effector Functions.. J Immunol.

[5] Skyler JS (2008). Update on worldwide efforts to prevent type 1 diabetes.. Ann N Y Acad Sci.

[6] Pescovitz MD, Greenbaum CJ, Krause-Steinrauf H, Becker DJ, Gitelman SE (2009). Rituximab, B-lymphocyte depletion, and preservation of beta-cell function.. N Engl J Med.

[7] Herold KC, Gitelman SE, Masharani U, Hagopian W, Bisikirska B (2005). A single course of anti-CD3 monoclonal antibody hOKT3gamma1(Ala-Ala) results in improvement in C-peptide responses and clinical parameters for at least 2 years after onset of type 1 diabetes.. Diabetes.

[8] Koulmanda M, Budo E, Bonner-Weir S, Qipo A, Putheti P (2007). Modification of adverse inflammation is required to cure new-onset type 1 diabetic hosts.. Proc Natl Acad Sci U S A.

[9] Koulmanda M, Bhasin M, Hoffman L, Fan Z, Qipo A (2008). Curative and beta cell regenerative effects of alpha1-antitrypsin treatment in autoimmune diabetic NOD mice.. Proc Natl Acad Sci U S A.

[10] Louvet C, Szot GL, Lang J, Lee MR, Martinier N (2008). Tyrosine kinase inhibitors reverse type 1 diabetes in nonobese diabetic mice.. Proc Natl Acad Sci U S A.

[11] Yang ZD, Chen M, Wu R, McDuffie M, Nadler JL (2002). The anti-inflammatory compound lisofylline prevents Type I diabetes in non-obese diabetic mice.. Diabetologia.

[12] Noorchashm H, Moore DJ, Lieu YK, Noorchashm N, Schlachterman A (1999). Contribution of the innate immune system to autoimmune diabetes: a role for the CR1/CR2 complement receptors.. Cell Immunol.

[13] Jensen-Urstad KJ, Reichard PG, Rosfors JS, Lindblad LE, Jensen-Urstad MT (1996). Early atherosclerosis is retarded by improved long-term blood glucose control in patients with IDDM.. Diabetes.

[14] Mangge H, Schauenstein K, Stroedter L, Griesl A, Maerz W (2004). Low grade inflammation in juvenile obesity and type 1 diabetes associated with early signs of atherosclerosis.. Exp Clin Endocrinol Diabetes.

[15] Wen L, Ley RE, Volchkov PY, Stranges PB, Avanesyan L (2008). Innate immunity and intestinal microbiota in the development of Type 1 diabetes.. Nature.

[16] Lee KU, Amano K, Yoon JW (1988). Evidence for initial involvement of macrophage in development of insulitis in NOD mice.. Diabetes.

[17] Foulis AK, McGill M, Farquharson MA (1991). Insulitis in type 1 (insulin-dependent) diabetes mellitus in man–macrophages, lymphocytes, and interferon-gamma containing cells.. J Pathol.

[18] Pasare C, Medzhitov R (2004). Toll-dependent control mechanisms of CD4 T cell activation.. Immunity.

[19] Thornley TB, Brehm MA, Markees TG, Shultz LD, Mordes JP (2006). TLR agonists abrogate costimulation blockade-induced prolongation of skin allografts.. J Immunol.

[20] D'Alise AM, Auyeung V, Feuerer M, Nishio J, Fontenot J (2008). The defect in T-cell regulation in NOD mice is an effect on the T-cell effectors.. Proc Natl Acad Sci U S A.

[21] You S, Belghith M, Cobbold S, Alyanakian MA, Gouarin C (2005). Autoimmune diabetes onset results from qualitative rather than quantitative age-dependent changes in pathogenic T-cells.. Diabetes.

[22] Eizirik DL, Mandrup-Poulsen T (2001). A choice of death–the signal-transduction of immune-mediated beta-cell apoptosis.. Diabetologia.

[23] Hayashi T, Faustman D (1999). NOD mice are defective in proteasome production and activation of NF-kappaB.. Mol Cell Biol.

[24] Eldor R, Yeffet A, Baum K, Doviner V, Amar D (2006). Conditional and specific NF-kappaB blockade protects pancreatic beta cells from diabetogenic agents.. Proc Natl Acad Sci U S A.

[25] Liuwantara D, Elliot M, Smith MW, Yam AO, Walters SN (2006). Nuclear factor-kappaB regulates beta-cell death: a critical role for A20 in beta-cell protection.. Diabetes.

[26] Poligone B, Weaver DJ, Sen P, Baldwin AS, Tisch R (2002). Elevated NF-kappaB activation in nonobese diabetic mouse dendritic cells results in enhanced APC function.. J Immunol.

[27] Sen P, Bhattacharyya S, Wallet M, Wong CP, Poligone B (2003). NF-kappa B hyperactivation has differential effects on the APC function of nonobese diabetic mouse macrophages.. J Immunol.

[28] Wheat W, Kupfer R, Gutches DG, Rayat GR, Beilke J (2004). Increased NF-kappa B activity in B cells and bone marrow-derived dendritic cells from NOD mice.. Eur J Immunol.

[29] Salojin KV, Zhang J, Madrenas J, Delovitch TL (1998). T-cell anergy and altered T-cell receptor signaling: effects on autoimmune disease.. Immunology Today.

[30] Noorchashm H, Moore DJ, Noto LE, Noorchashm N, Reed AJ (2000). Impaired CD4 T cell activation due to reliance upon B cell-mediated costimulation in nonobese diabetic (NOD) mice.. J Immunol.

[31] Greeley SA, Moore DJ, Noorchashm H, Noto LE, Rostami SY (2001). Impaired activation of islet-reactive CD4 T cells in pancreatic lymph nodes of B cell-deficient nonobese diabetic mice.. J Immunol.

[32] Arreaza G, Salojin K, Yang W, Zhang J, Gill B (2003). Deficient activation and resistance to activation-induced apoptosis of CD8+ T cells is associated with defective peripheral tolerance in nonobese diabetic mice.. Clin Immunol.

[33] Hussain S, Salojin KV, Delovitch TL (2004). Hyperresponsiveness, resistance to B-cell receptor-dependent activation-induced cell death, and accumulation of hyperactivated B-cells in islets is associated with the onset of insulitis but not type 1 diabetes.. Diabetes.

[34] Hawiger J (2001). Innate immunity and inflammation: a transcriptional paradigm.. Immunol Res.

[35] Hussain S, Delovitch TL (2005). Dysregulated B7-1 and B7-2 expression on nonobese diabetic mouse B cells is associated with increased T cell costimulation and the development of insulitis.. J Immunol.

[36] Lin YZ, Yao SY, Veach RA, Torgerson TR, Hawiger J (1995). Inhibition of nuclear translocation of transcription factor NF-kappa B by a synthetic peptide containing a cell membrane-permeable motif and nuclear localization sequence.. J Biol Chem.

[37] Torgerson TR, Colosia AD, Donahue JP, Lin YZ, Hawiger J (1998). Regulation of NF-kappa B, AP-1, NFAT, and STAT1 nuclear import in T lymphocytes by noninvasive delivery of peptide carrying the nuclear localization sequence of NF-kappa B p50.. J Immunol.

[38] Liu X, Robinson D, Veach RA, Liu D, Timmons S (2000). Peptide-directed suppression of a pro-inflammatory cytokine response.. J Biol Chem.

[39] Liu D, Li C, Chen Y, Burnett C, Liu XY (2004). Nuclear import of proinflammatory transcription factors is required for massive liver apoptosis induced by bacterial lipopolysaccharide.. J Biol Chem.

[40] Liu D, Zienkiewicz J, DiGiandomenico A, Hawiger J (2009). Suppression of acute lung inflammation by intracellular peptide delivery of a nuclear import inhibitor.. Mol Ther.

[41] Baxter AG, Mandel TE (1991). Accelerated diabetes in non-obese diabetic (NOD) mice differing in incidence of spontaneous disease.. Clin Exp Immunol.

[42] Chatenoud L, Thervet E, Primo J, Bach JF (1994). Anti-CD3 antibody induces long-term remission of overt autoimmunity in nonobese diabetic mice.. Proc Natl Acad Sci U S A.

[43] Ablamunits V, Quintana F, Reshef T, Elias D, Cohen IR (1999). Acceleration of autoimmune diabetes by cyclophosphamide is associated with an enhanced IFN-gamma secretion pathway.. J Autoimmun.

[44] Matos M, Park R, Mathis D, Benoist C (2004). Progression to islet destruction in a cyclophosphamide-induced transgenic model: a microarray overview.. Diabetes.

[45] Signore A, Procaccini E, Toscano AM, Ferretti E, Williams AJ (1994). Histological study of pancreatic beta-cell loss in relation to the insulitis process in the non-obese diabetic mouse.. Histochemistry.

[46] Liu D, Liu XY, Robinson D, Burnett C, Jackson C (2004). Suppression of Staphylococcal Enterotoxin B-induced Toxicity by a Nuclear Import Inhibitor.. J Biol Chem.

[47] Suk K, Kim S, Kim YH, Kim KA, Chang I (2001). IFN-gamma/TNF-alpha synergism as the final effector in autoimmune diabetes: a key role for STAT1/IFN regulatory factor-1 pathway in pancreatic beta cell death.. J Immunol.

[48] Papaccio G, Graziano A, D'Aquino R, Valiante S, Naro F (2005). A biphasic role of nuclear transcription factor (NF)-kappaB in the islet beta-cell apoptosis induced by interleukin (IL)-1beta.. J Cell Physiol.

[49] Rasschaert J, Ladriere L, Urbain M, Dogusan Z, Katabua B (2005). Toll-like receptor 3 and STAT-1 contribute to double-stranded RNA+ interferon-gamma-induced apoptosis in primary pancreatic beta-cells.. J Biol Chem.

[50] Theodore M, Kawai Y, Yang J, Kleshchenko Y, Reddy SP (2008). Multiple nuclear localization signals function in the nuclear import of the transcription factor Nrf2.. J Biol Chem.

[51] Veach RA, Liu D, Yao S, Chen Y, Liu XY (2004). Receptor/transporter-independent targeting of functional peptides across the plasma membrane.. J Biol Chem.

[52] Ramamoorthy A, Kandasamy SK, Lee DK, Kidambi S, Larson RG (2007). Structure, topology, and tilt of cell-signaling peptides containing nuclear localization sequences in membrane bilayers determined by solid-state NMR and molecular dynamics simulation studies.. Biochemistry.

[53] Webb SR, Hutchinson J, Hayden K, Sprent J (1994). Expansion/deletion of mature T cells exposed to endogenous superantigens in vivo.. J Immunol.

[54] Serreze DV, Chapman HD, Post CM, Johnson EA, Suarez-Pinzon WL (2001). Th1 to Th2 cytokine shifts in nonobese diabetic mice: sometimes an outcome, rather than the cause, of diabetes resistance elicited by immunostimulation.. J Immunol.

[55] Li FX, Zhu JW, Tessem JS, Beilke J, Varella-Garcia M (2003). The development of diabetes in E2f1/E2f2 mutant mice reveals important roles for bone marrow-derived cells in preventing islet cell loss.. Proc Natl Acad Sci U S A.

[56] Kendall PL, Woodward EJ, Hulbert C, Thomas JW (2004). Peritoneal B cells govern the outcome of diabetes in non-obese diabetic mice.. Eur J Immunol.

[57] Hoglund P, Mintern J, Waltzinger C, Heath W, Benoist C (1999). Initiation of autoimmune diabetes by developmentally regulated presentation of islet cell antigens in the pancreatic lymph nodes.. J Exp Med.

